# Employment status and occupational level of adult survivors of childhood cancer in Great Britain: The British childhood cancer survivor study

**DOI:** 10.1002/ijc.30696

**Published:** 2017-04-07

**Authors:** Clare Frobisher, Emma R Lancashire, Helen Jenkinson, David L Winter, Julie Kelly, Raoul C Reulen, Michael M Hawkins, On behalf of the British Childhood Cancer Survivor Study (BCCSS) Steering Group

**Affiliations:** ^1^ Department of Public HealthEpidemiology and Biostatistics, Centre for Childhood Cancer Survivor Studies, Institute of Applied Health Research, University of BirminghamBirminghamB15 2TTUnited Kingdom; ^2^ Department of Public HealthEpidemiology and Biostatistics, WAVES study office, Institute of Applied Health Research, University of BirminghamBirminghamB15 2TTUnited Kingdom; ^3^ Department of OncologyBirmingham Children's Hospital, NHS Foundation TrustSteelhouse LaneBirminghamB4 6NHUnited Kingdom

**Keywords:** childhood cancer, survivorship, employment, occupation, economic status, social outcome

## Abstract

The British Childhood Cancer Survivor Study (BCCSS) provides the first detailed investigation of employment and occupation to be undertaken in a large population‐based cohort. Previous studies have been limited by design issues such as using small numbers of survivors with specific diagnoses, and involved limited assessment of employment status and occupational level. The BCCSS includes 17,981 5‐year survivors of childhood cancer. Employment status and occupational level were ascertained by questionnaire from eligible survivors (*n* = 14,836). Multivariate logistic regression was used to explore factors associated with employment and occupation, and to compare survivors to their demographic peers in the general population. Employment status was available for 10,257 survivors. Gender, current age, cancer type, radiotherapy, age at diagnosis and epilepsy were consistently associated with being: employed; unable to work; in managerial or non‐manual occupations. Overall, survivors were less likely to be working than expected (OR (99% CI): 0.89 (0.81–0.98)), and this deficit was greatest for irradiated CNS neoplasm survivors (0.34 (0.28–0.41)). Compared to the general population, survivors were fivefold more likely to be unable to work due to illness/disability; the excess was 15‐fold among CNS neoplasm survivors treated with radiotherapy. Overall survivors were less likely to be in managerial occupations than expected (0.85 (0.77–0.94)). However, bone sarcoma survivors were more likely to be in these occupations than expected (1.37 (1.01–1.85)) and also similarly for non‐manual occupations (1.90 (1.37–2.62)). Survivors of retinoblastoma (1.55 (1.20–2.01)) and ‘other’ neoplasm group (1.62 (1.30–2.03)) were also more likely to be in non‐manual occupations than expected.

## Abstract 2

**What's new?**

Employment and job satisfaction contribute to individual wellbeing and performance in modern society. For survivors of childhood cancer, however, poor health can significantly hinder the ability to work, the present study suggests. Using data from the British Childhood Cancer Survivor Study, the authors show that compared with the general population, survivors of childhood cancer were less likely to be working, with work activity limited particularly by illness and disability. Impacts were greatest for survivors of central nervous system neoplasms treated by radiotherapy. The findings help identify factors that could be targeted to maximize survivors' employment potential.

## Introduction

Employment status and occupational level are measures of an adult's performance in today's competitive society. As the population of individuals diagnosed with cancer in childhood and surviving into adulthood continues to expand,^1^, ^2^ it becomes increasingly important to investigate such outcomes among this group of adult survivors of childhood cancer and, in particular, determine how their performance compares to that of the general population.

A meta‐analysis investigating employment status in childhood cancer survivors reported that previous studies were generally small, had relatively short lengths of follow‐up or were not population‐based.^3^ In addition, few studies have investigated the specific reasons why survivors are not in current employment^4^, ^5^ or considered occupational level in a large‐scale study.^5^, ^6^ The British Childhood Cancer Survivor Study (BCCSS) has enabled the first detailed investigation of employment status, together with a consideration of occupational level, to be undertaken in a large population‐based cohort with a considerable period of follow‐up, containing most adult survivors of childhood cancer in Britain. General population comparisons have been undertaken and factors influencing employment status and occupational level have been determined.

## Material and Methods

The BCCSS, described in detail elsewhere,^7^ is a cohort of five‐year survivors of childhood cancer diagnosed in Britain between 1940 and 1991, and identified through the National Registry of Childhood Tumours. A study questionnaire was sent, via the general practitioner, to survivors who were resident in Britain and aged at least 16 years. Of 14,836 eligible survivors, 70.7% (10,488) returned a completed questionnaire.^7^ The median age at questionnaire completion was 28.9 years (range 16.0–74.2 years); 29.4% of the survivors were aged 35 years or older at questionnaire completion. Appropriate ethical approval was obtained for the study (West Midlands Multi‐centre Research Ethics Committee approval followed by approval from all Local Research Ethics Committees nationally (212 in total)).

For the external comparisons with the general population, data were taken from the General Household Survey (GHS).^8^, ^9^ The GHS was an annual survey carried out by the Office for National Statistics (ONS) which ran from 1971 to 2012 to collect varying information from people living in private households in Great Britain. The GHS sampled approximately 13,000 addresses each year and aimed to interview all adults (16 years or over) at every household at the sampled address. We chose the GHS database for the year for which we had the largest proportion (50.4%) of BCCSS questionnaires completed and returned. The questions included in the employment section of the BCCSS questionnaire obtained comparable information to that collected in the GHS. The BCCSS questionnaire can be viewed in full at: http://www.birmingham.ac.uk/research/activity/mds/projects/HaPS/PHEB/CCCSS/bccss/documents.aspx

**Figure 1:**
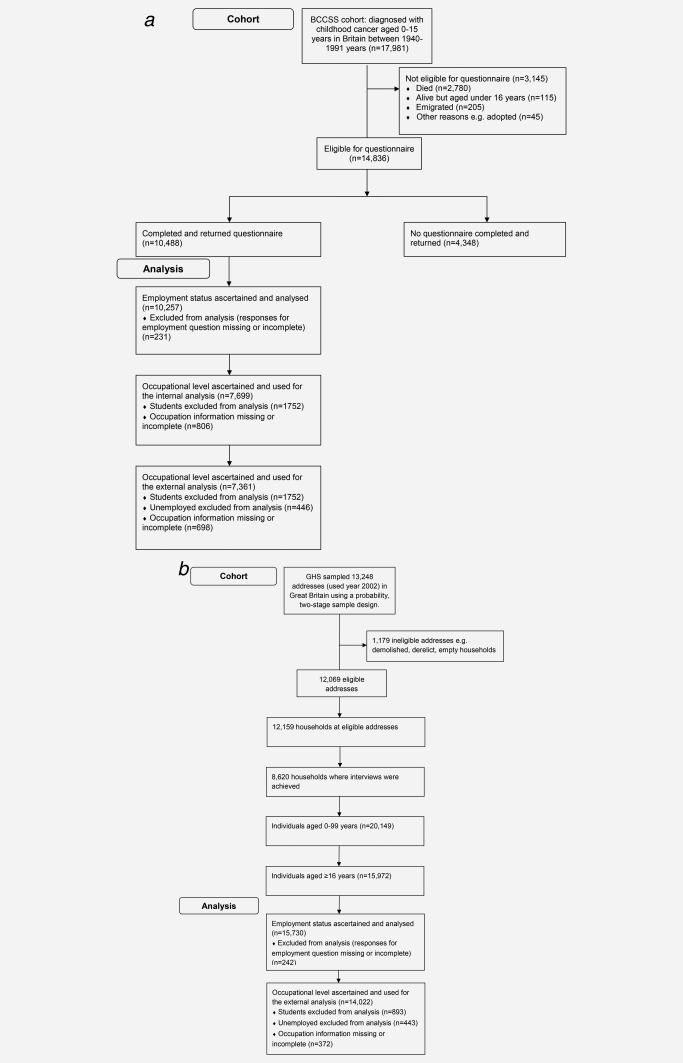
(*a*) Flow chart detailing the numbers involved at each stage from the BCCSS. (*b*) Flow chart detailing the numbers involved at each stage from the General Household Survey (GHS) for the year 2002.

Employment status was available for 10,257 survivors and 15,730 GHS participants aged at least 16 years (Fig. 1), from a closed‐response to the question: ‘What is your current employment status? Working full‐time or part‐time; unemployed and looking for work; unable to work due to illness or disability; caring for home or family not seeking paid work; student; retired. Survivors were classified as either YES or NO in relation to each of these six possibilities and each of these six binary outcomes formed the outcome variable for the logistic regression in Tables 1, 2 and in Supporting Information e‐table 2. However, retired individuals (*n* = 68) were not included in the analysis as the numbers were too small for meaningful analysis.

**Table 1:** Frequency of survivors for the outcomes being: employed, unemployed; and unable to work due to illness and disability, and the corresponding ORs (99% CIs) from multivariable logistic regression for these outcomes with selected demographic, cancer and health related factors in the childhood cancer survivors

		Survivors who were employed			Survivors who were unemployed and looking for work			Survivors unable to work due to illness or disability		
Factor	Total number of survivors	% of survivors who were employed	Adjusted odds ratio for being employed *vs*. not^a^	99% CI	% of survivors who were unemployed and looking for work	Adjusted odds ratio for being unemployed *vs*. not^a^	99% CI	% of survivors unable to work due to illness/disability	Adjusted odds ratio for being unable to work due to illness/disability *vs*. not^a^	99% CI
**Gender**										
Male	5256	67.2	1.00		5.2	1.00		9.4	1.00	
Female	5001	57.7	0.58	0.51–0.66	3.4	0.63	0.48–0.82	12.0	1.33	1.09–1.62
*P*_heterogeneity_				<0.001			<0.001			<0.001
**Current age (at questionnaire completion in years)**										
16–19	1991	24.9	1.00		5.2	1.00		2.7	1.00	
20–24	1712	63.4	5.64	4.60–6.92	6.7	1.32	0.90–1.94	8.4	4.35	2.65–7.12
25–29	1877	76.7	11.76	9.42–14.68	4.1	0.74	0.47–1.15	10.9	5.43	3.35–8.82
30–34	1668	74.8	10.87	8.64–13.67	4.0	0.72	0.46–1.15	12.3	6.31	3.87–10.30
35–39	1255	75.4	11.44	8.88–14.73	3.0	0.62	0.36–1.06	13.2	6.81	4.10–11.31
40–44	744	71.0	9.10	6.82–12.14	3.0	0.51	0.26–1.01	18.2	10.54	6.23–17.85
45–49	485	73.0	10.50	7.41–14.87	2.9	0.57	0.25–1.27	18.6	10.14	5.69–18.05
50–54	333	67.9	7.96	5.43–11.67	2.4	0.44	0.15–1.26	18.9	10.71	5.77–19.89
≥ 55	192	47.9	3.07	1.94–4.88	1.6	0.11	0.01–1.53	18.8	12.27	5.93–25.39
*P*_heterogeneity_				<0.001			<0.001			<0.001
*P*_linear trend_ (*P*_non‐linearity_)				<0.001 (<0.001)			<0.001 (0.072)			<0.001 (<0.001)
**Cancer type**										
CNS neoplasm	2153	51.5	1.00		5.2	1.00		26.1	1.00	
Leukaemia	2819	60.0	1.57	1.28–1.92	5.0	0.92	0.61–1.38	6.4	0.51	0.38–0.69
Hodgkin's lymphoma	724	81.1	2.03	1.49–2.78	3.7	0.84	0.45–1.58	4.8	0.28	0.16–0.47
Non‐hodgkin's lymphoma	530	74.5	1.79	1.29–2.48	3.4	0.64	0.31–1.34	6.0	0.43	0.25–0.73
Neuroblastoma	420	54.0	1.46	1.01–2.10	4.5	1.00	0.46–2.15	5.7	0.43	0.22–0.81
Retinoblastoma	692	62.7	2.38	1.72–3.28	5.8	1.32	0.72–2.41	7.1	0.21	0.13–0.35
Wilms' tumour	954	63.0	1.53	1.17–2.00	3.5	0.74	0.41–1.32	5.4	0.41	0.26–0.65
Bone sarcoma	389	72.8	1.29	0.91–1.85	1.8	0.45	0.16–1.28	10.8	0.78	0.48–1.30
Soft tissue sarcomas	706	67.1	1.60	1.20–2.13	3.5	0.74	0.39–1.40	8.6	0.60	0.39–0.91
Other neoplasm	870	70.5	1.92	1.46–2.51	3.0	0.67	0.35–1.25	6.6	0.37	0.24–0.57
*P*_heterogeneity_				<0.001			0.155			<0.001
**Treatment**										
Surgery No	3355	70.5	1.00		4.5	1.00		9.2	1.00	
Yes	4185	68.1	0.79	0.64–0.96	3.7	0.94	0.61–1.46	15.0	1.46	1.09–1.94
*P*_heterogeneity_				0.002			0.728			<0.001
Radiotherapy (RT) No RT	2176	69.8	1.00		3.3	1.00		10.4	1.00	
Non‐cranial RT	2231	76.6	0.96	0.77–1.19	3.0	1.10	0.67–1.81	9.6	1.49	1.08–2.05
Cranial RT	2909	62.6	0.62	0.50–0.77	5.5	1.38	0.85–2.22	16.5	2.51	1.84–3.41
*P*_heterogeneity_				<0.001			0.200			<0.001
Chemotherapy No	3268	66.0	1.00		3.6	1.00		17.1	1.00	
Yes	3834	71.4	1.09	0.88–1.34	4.4	0.94	0.60–1.46	8.0	0.82	0.61–1.10
*P*_heterogeneity_				0.290			0.701			0.086
**Age at cancer diagnosis (years)**										
0	834	52.6	1.00		4.1	1.00		7.6	1.00	
1–4	3900	53.9	1.07	0.83–1.40	5.0	1.37	0.77–2.41	9.1	1.10	0.69–1.73
5–9	2719	66.8	1.41	1.05–1.88	4.8	1.43	0.77–2.69	11.6	0.79	0.48–1.30
10–14	2804	73.4	1.43	1.05–1.94	3.1	1.20	0.61–2.36	13.0	0.70	0.42–1.16
*P*_heterogeneity_				<0.001			0.329			<0.001
*P*_linear trend_ (*P*_non‐linearity_)				<0.001 (0.076)			0.939 (0.180)			<0.001 (0.153)
**Second primary tumour diagnosed**										
No	9648	62.9	1.00		4.4	1.00		10.0	1.00	
Yes	609	57.6	0.68	0.52–0.88	3.9	1.06	0.58–1.93	21.7	1.63	1.17–2.27
*P*_heterogeneity_				<0.001			0.806			<0.001
**Epilepsy or repeated seizures/fits diagnosed**										
No	9030	65.8	1.00		4.2	1.00		7.3	1.00	
Yes	980	38.2	0.33	0.27–0.42	5.4	1.31	0.85–2.01	38.1	4.89	3.84–6.23
*P*_heterogeneity_				<0.001			0.111			<0.001
**At least one hearing problem diagnosed**										
No	9002	63.9	1.00		4.3	1.00		9.1	1.00	
Yes	965	53.1	0.75	0.61–0.93	4.4	1.03	0.65–1.63	22.9	1.77	1.35–2.32
*P*_heterogeneity_				<0.001			0.880			<0.001
**At least one vision problem diagnosed**										
No	8684	65.5	1.00		4.1	1.00		8.0	1.00	
Yes	1297	45.6	0.44	0.36–0.54	5.9	1.34	0.89–2.01	26.4	3.00	2.33–3.86
*P*_heterogeneity_				<0.001			0.069			<0.001
**Recurrence**										
No	8848	64.1	1.00		4.2	1.00		9.5	1.00	
Yes	1334	53.3	0.69	0.58–0.84	5.0	1.07	0.73–1.57	17.7	1.72	1.33–2.22
*P*_heterogeneity_				<0.001			0.649			<0.001

a For all factors in the above table, with the exception of the treatment factors (surgery, chemotherapy and radiotherapy), the multivariable logistic regression included all factors without surgery, chemotherapy and radiotherapy and the resulting odds ratios and *p* values are from this model. For the treatment factors, the multivariable logistic regression included all factors with the exception of cancer type and the resulting odds ratios and *p* values for the treatment factors are from this model.

**Table 2:** Odds ratios (OR) and 99% confidence intervals (CI) for adult survivors of childhood cancer who were: employed; unemployed; unable to work due to illness or disability; a student; caring for home or family compared to the general population of Britain, overall and by childhood cancer type, survivors treated with and without radiotherapy are considered separately for CNS neoplasms^a^

	Employed			Unemployed and looking for work			Unable to work due to illness or disability			Student			Caring for home or family		
Cancer type^a^	OR	(99% CI)	*p* value	OR	(99% CI)	*p* value	OR	(99% CI)	*p* value	OR	(99% CI)	*p* value	OR	(99% CI)	*p* value
All cancer types	0.89	(0.81–0.98)	0.002	0.89	(0.72–1.09)	0.137	4.99	(4.06–6.13)	<0.0005	1.13	(0.97–1.32)	0.043	0.63	(0.53–0.74)	<0.0005
Leukaemia^c^ RT=Yes	1.13	(0.96–1.34)	0.055	0.99	(0.72–1.37)	0.955	4.41	(3.07–6.32)	<0.0005	0.78	(0.61–0.99)	0.007^d^	0.60	(0.45–0.82)	<0.0005
Hodgkin's lymphoma	1.55	(1.18–2.04)	<0.0005	0.84	(0.50–1.44)	0.413	1.49	(0.92–2.41)	0.033	0.81	(0.50–1.33)	0.272	0.63	(0.37–1.06)	0.022
Non‐Hodgkin's lymphoma	1.39	(1.04–1.85)	0.004	0.71	(0.37–1.34)	0.162	2.24	(1.34–3.75)	<0.0005	0.88	(0.59–1.30)	0.395	0.76	(0.42–1.37)	0.235
CNS neoplasm^b^ RT=Yes	0.34	(0.28–0.41)	<0.0005	1.50	(1.04–2.18)	0.005^d^	15.40	(11.94–19.85)	<0.0005	0.69	(0.45–1.04)	0.020	0.45	(0.29–0.71)	<0.0005
RT=No	0.64	(0.49–0.82)	<0.0005	0.74	(0.40–1.38)	0.214	8.29	(6.08–11.31)	<0.0005	0.93	(0.58–1.49)	0.679	0.86	(0.56–1.33)	0.373
Neuroblastoma	0.83	(0.62–1.11)	0.098	0.86	(0.45–1.63)	0.537	2.93	(1.62–5.30)	<0.0005	1.64	(1.15–2.34)	<0.0005	0.65	(0.34–1.24)	0.087
Retinoblastoma	0.94	(0.74–1.20)	0.534	1.30	(0.83–2.05)	0.137	2.69	(1.76–4.11)	<0.0005	1.50	(1.10–2.05)	0.001	0.62	(0.39–1.00)	0.010
Wilms tumour	1.04	(0.84–1.27)	0.668	0.67	(0.41–1.09)	0.036	2.67	(1.73–4.11)	<0.0005	1.40	(1.07–1.84)	0.001	0.65	(0.43–0.98)	0.007^d^
Bone sarcomas	1.16	(0.82–1.66)	0.274	0.41	(0.15–1.12)	0.023	3.98	(2.49–6.38)	<0.0005	0.59	(0.29–1.18)	0.049	0.92	(0.55–1.56)	0.694
Soft tissue sarcomas	1.14	(0.89–1.45)	0.185	0.74	(0.43–1.29)	0.164	3.41	(2.29–5.08)	<0.0005	1.15	(0.82–1.61)	0.280	0.54	(0.32–0.92)	0.003
Other neoplasm	1.30	(1.04–1.64)	0.003	0.65	(0.38–1.11)	0.040	2.59	(1.74–3.87)	<0.0005	1.06	(0.77–1.45)	0.659	0.69	(0.46–1.02)	0.013

a Population data from the GHS (2002) was used for the reference group.

b Excludes survivors for whom no record is available regarding radiotherapy.

c There were insufficient leukaemia survivors known to have been unexposed to cranial irradiation for separate meaningful assessment.

d On using the False Discovery Rate method the corrected *p* values indicated that the null hypothesis was not rejected for these particular tests.

Details relating to current employment, otherwise most recent employment, were obtained to enable individuals to be classified into defined occupational levels (managerial or professional, intermediate, routine or manual) using the National Statistics Socio‐economic Classification (NS‐SEC). NS‐SEC is an occupational‐based classification used in Britain for official statistics.^9^ Individuals who were a student (1,752 survivors, 893 GHS participants) were excluded from all analyses relating to occupation as many would never have been in employment and for those who provided employment information it could potentially be misleading in relation to occupational level they might ultimately achieve. For unemployed individuals who participated in the GHS, occupational level (based on most recent employment) is only available for individuals who were unemployed for a period of <12 months; whereas occupational level was available for all unemployed childhood cancer survivors irrespective of the period of unemployment. Therefore, to ensure comparability between the two datasets, unemployed individuals were excluded from all external analyses relating to occupational level (446 survivors, 443 GHS participants). Two binary outcomes were considered in relation to occupational level: those classified to a managerial or professional occupation and the remainder; those classified to non‐manual occupations (including managerial/professional and intermediate occupations) and the remainder. Figure 1 details the numbers used at each stage from the BCCSS and the GHS.

For both employment status and occupational level several potential explanatory risk factors were identified *a priori*: sex; age at questionnaire completion; childhood cancer type; chemotherapy (yes, no); surgery (yes, no); radiotherapy (RT classified into: cranial RT, non‐cranial RT, no RT); age at diagnosis; whether diagnosed with a second primary tumour (SPT); whether diagnosed with epilepsy, repeated seizures or fits; whether diagnosed with at least one hearing problem (hearing loss requiring a hearing aid, deafness in one or both ears not corrected by a hearing aid, complete deafness in either ear or problems hearing sounds in crowds); whether diagnosed with at least one serious visual problem (registered as blind or trouble seeing with one or both eyes even when wearing glasses); whether or not the survivor reported a recurrence of their original tumour.

### Statistical analysis

For the internal analysis potential explanatory risk factors for employment status and occupational level were investigated using multivariate logistic regression that included all factors identified above with the exception of the treatment factors (surgery, radiotherapy and chemotherapy) due to the strong relationship between treatment and cancer type. To investigate the effect of treatment all factors above with the exception of cancer type were included in the model. Tests for heterogeneity (based on the likelihood‐ratio statistic) having adjusted for the other factors were undertaken. Likelihood‐ratio tests for both linear trend and departure from linearity were also performed for factors where the categories had at least ordinal properties.

For the external analysis, we compared both employment status and occupational level within the survivor population to that expected from the general population, logistic regression adjusting for age and sex was used. To take into account household clustering in the GHS, a logistic regression model with a generalised estimating equation modification was used. A weighting factor was used for the population data, both to compensate for non‐response in the GHS and to match the GHS sample to known population distributions.^9^

For all statistical tests we have presented the *p* values on adjustment for identified confounders and statistical significance was taken at the 1% level (2‐sided test) due to the large sample size. We have also performed the Benjamini‐Hochberg^10^ correction for the false discovery rate (FDR) in multiple comparisons setting the point‐wise threshold for the correction at 0.01. If previously using the uncorrected *p* values the null hypothesis was rejected but on adjustment using the FDR the null hypothesis was not rejected then this was noted in the table of results and discussed in the Results and Discussion sections. All analyses were carried out using Stata (version 14; Stata Corp., College Station, TX).

## Results

### Internal analysis

Of the 10,257 survivors for whom employment status was available: 63% were working; 17% were students; 11% were unable to work due to illness/disability; 5% were caring for home/family; 4% were unemployed and looking for work; 1% were retired (Supporting Information etable 1 ‐ appendix). When occupational level was considered 31% of survivors were classified to managerial/professional occupations, 25% to intermediate and 44% to manual/routine occupations.

### Factors influencing each employment status

#### Employed

Factors found to influence whether a survivor was working included: sex; current age; childhood cancer type; surgery; radiotherapy; age at diagnosis; diagnosis of a SPT, epilepsy, hearing problems, visual problems or recurrence (Table 1). Females were less likely (odds ratio [OR] (99% CI): 0.58 (0.51–0.66)) to be working than males. Likelihood of being employed initially increased with age and then plateaued but declined after 45–49 years. All survivors with the exception of those diagnosed with a bone sarcoma were more likely to be in employment than CNS neoplasm survivors. Survivors treated with surgery were less likely (OR (99% CI): 0.79 (0.64–0.96)) to be employed than those who did not have surgery treatment. Cranially irradiated survivors were less likely (OR (99% CI): 0.62 (0.50–0.77)) to be in employment than those who did not receive radiotherapy. Likelihood of being in employment increased with age at diagnosis. Individuals diagnosed with a SPT (OR (99% CI): 0.68 (0.52–0.88)), epilepsy (0.33 (0.27–0.42)), a hearing problem (0.75 (0.61–0.93)), visual problem (0.44 (0.36–0.54)) or recurrence (0.69 (0.58–0.84)) were all less likely to be in employment than those without such medical history.

#### Unemployed and looking for work

The only factors found to influence unemployment in survivors were sex and current age (Table 1). Females were less likely (OR (99% CI): 0.63 (0.48–0.82)) to be unemployed than males. After an initial increase in survivors being unemployed with age, the likelihood for this outcome decreased with increasing age.

#### Unable to work due to illness/disability

Sex, current age, cancer type, surgery, radiotherapy, age at diagnosis, diagnosis of a SPT, epilepsy, hearing problems, visual problems and recurrence were all found to influence the likelihood of a survivor being unable to work due to illness/disability (Table 1). Females were more likely (OR (99% CI): 1.33 (1.09–1.62)) to be reporting this outcome than males. The likelihood of this outcome increased with current age to 40–44 years after which the likelihood plateaus. With regard to cancer type, the majority of survivors were less likely to be unable to work due to illness/disability than CNS neoplasm survivors, particularly retinoblastoma survivors (OR (99% CI): 0.21 (0.13–0.35)); however, for bone sarcoma survivors there was no evidence of a difference. Survivors treated with surgery were more likely (OR (99% CI): 1.46 (1.09–1.94)) to be unable to work due to illness/disability than those not so treated. The OR of being unable to work due to illness/disability was more than doubled (OR (99% CI): 2.51 (1.84–3.41)) for survivors treated with cranial radiotherapy compared with those not treated with radiotherapy. The likelihood of the outcome decreased with increasing age at diagnosis. Survivors with a SPT (OR (99% CI): 1.63 (1.17–2.27)), epilepsy (4.89 (3.84–6.23)), a hearing (1.77 (1.35–2.32)) or sight problem (3.00 (2.33–3.86)) or a recurrence (1.72 (1.33–2.22)) all had increased OR for being unable to work due to illness/disability compared to those without such medical history.

#### Student

Factors influencing whether a survivor was a student were current age and age at diagnosis (Supporting Information eTable 2 ‐ Appendix). The likelihood of being a student declined in relation to increases in both of these ages.

#### Caring for home or family and not seeking paid work

Only sex and current age were significantly associated with caring for home/family (Supporting Information eTable 2 ‐ Appendix). Females were considerably more likely (OR (99% CI): 18.25 (10.78–30.92)) to be caring for home/family than males. Up to 34 years the likelihood of caring for home/family increased with age, thereafter it plateaued.

### Factors influencing each occupational level outcome

#### Managerial/professional

The likelihood of survivors being classified to managerial/professional occupations was associated with: sex; current age; cancer type; radiotherapy; chemotherapy: age at diagnosis and diagnosis with epilepsy (Table 3). Females were less likely (OR (99% CI): 0.80 (0.69–0.91)) than males to be classified as managerial/professional. The likelihood of being classified as managerial/professional increased with age to 29 years, and thereafter, it plateaued until the age of 45 years when it decreased again. All diagnoses were more likely to be classified as managerial/professional occupations than survivors of a CNS neoplasm; in particular for retinoblastoma survivors (OR (99% CI): 2.79 (1.97–3.97)). Those treated with cranial irradiation were less likely (OR (99% CI): 0.65 (0.52–0.82)) than those who received no radiotherapy to be managerial/professional. Survivors who received chemotherapy were more likely (OR (99% CI): 1.27 (1.04–1.55)) to be managerial/professional than those not so treated. The likelihood of being in a managerial/professional occupation increased with increasing age at diagnosis. The odds for being in a managerial/professional occupation for survivors with epilepsy was approximately half (OR (99% CI): 0.57 (0.42–0.78)) that of those without such a diagnosis.

**Table 3:** Frequency of survivors for the outcomes being classified to: managerial/professional occupation; and to non‐manual occupation, and the corresponding ORs (99% CIs) from multivariable logistic regression for these outcomes with selected demographic, cancer and health related factors in the childhood cancer survivors

		Managerial/professional occupational level			Non‐manual occupational level		
Factor	Total number of survivors^a^	% of survivors who were classified to this level	Adjusted odds ratio for being classified to this level *vs*. a lower level^b^	99% CI	% of survivors who were classified to this level	Adjusted odds ratio for being classified to this level *vs*. a manual occupational level^b^	99% CI
**Gender**							
Male	4014	32.5	1.00		53.2	1.00	
Female	3685	29.0	0.80	0.69–0.91	58.1	1.15	1.01–1.31
*P*_heterogeneity_				<0.001			0.005
**Current age (at questionnaire completion in years)**							
16–19	550	3.8	1.00		22.7	1.00	
20–24	1234	23.3	7.73	4.17–14.32	49.0	3.27	2.39–4.48
25–29	1640	36.5	13.45	7.32–24.71	60.2	4.77	3.50–6.50
30–34	1493	34.2	12.55	6.81–23.11	59.8	4.69	3.42–6.42
35–39	1156	34.9	12.60	6.80–23.36	59.7	4.52	3.26–6.26
40–44	680	37.4	14.04	7.45–26.44	61.8	4.77	3.33–6.82
45–49	448	33.5	10.77	5.59–20.76	61.2	4.41	2.98–6.52
50–54	315	28.6	8.74	4.40–17.38	56.8	3.47	2.26–5.31
≥ 55	183	31.7	10.49	4.98–22.09	56.8	3.69	2.20–6.21
*P*_heterogeneity_				<0.001			<0.001
*P*_linear trend_ (*P*_non‐linearity_)				<0.001 (<0.001)			<0.001 (<0.001)
**Cancer type**							
CNS neoplasm	1528	21.9	1.00		48.4	1.00	
Leukaemia	1943	25.0	1.34	1.06–1.71	47.5	1.15	0.93–1.42
Hodgkin's lymphoma	660	37.4	1.59	1.20–2.12	57.9	1.23	0.94–1.60
Non‐Hodgkin's lymphoma	450	34.9	1.66	1.20–2.28	59.1	1.50	1.11–2.02
Neuroblastoma	270	33.7	2.30	1.49–3.54	57.8	2.01	1.34–3.02
Retinoblastoma	528	38.1	2.79	1.97–3.97	65.2	2.36	1.69–3.29
Wilms' tumour	706	32.9	2.09	1.54–2.84	58.8	1.95	1.48–2.57
Bone sarcoma	347	42.7	2.20	1.56–3.10	71.8	2.43	1.69–3.48
Soft tissue sarcomas	557	37.9	2.12	1.57–2.86	59.6	1.69	1.27–2.25
Other neoplasm	710	37.5	1.98	1.50–2.62	66.3	2.07	1.58–2.71
*P*_heterogeneity_				<0.001			<0.001
**Treatment**							
Surgery No	2753	29.7	1.00		53.7	1.00	
Yes	3567	34.1	1.13	0.93–1.36	59.9	1.10	0.91–1.32
*P*_heterogeneity_				0.108			0.207
Radiotherapy (RT) No RT	1836	34.2	1.00		60.6	1.00	
Non‐cranial RT	2037	39.7	1.16	0.96–1.41	64.9	1.14	0.94–1.39
Cranial RT	2250	23.6	0.65	0.52–0.82	47.5	0.63	0.52–0.78
*P*_heterogeneity_				<0.001			<0.001
Chemotherapy No	2765	31.7	1.00		58.8	1.00	
Yes	3164	32.3	1.27	1.04–1.55	55.8	1.16	0.95–1.40
*P*_heterogeneity_				0.002			0.054
**Age at cancer diagnosis (years)**							
0	524	36.3	1.00		60.1	1.00	
1–4	2484	25.0	0.79	0.59–1.08	49.2	0.88	0.65–1.18
5–9	2174	28.0	1.00	0.72–1.40	52.6	1.10	0.80–1.51
10–14	2517	37.9	1.34	0.95–1.87	63.4	1.47	1.06–2.04
*P*_heterogeneity_				<0.001			<0.001
*P*_linear trend_ (*P*_non‐linearity_)				<0.001 (<0.001)			<0.001 (0.005)^c^
**Second primary tumour diagnosed**							
No	7209	30.7	1.00		55.3	1.00	
Yes	490	32.2	0.99	0.75–1.31	59.2	1.06	0.81–1.38
*P*_heterogeneity_				0.922			0.595
**Epilepsy or repeated seizures/fits diagnosed**							
No	6982	32.1	1.00		56.6	1.00	
Yes	586	18.6	0.57	0.42–0.78	45.4	0.70	0.54–0.89
*P*_heterogeneity_				<0.001			0.002
**At least one hearing problem diagnosed**							
No	6805	31.6	1.00		56.3	1.00	
Yes	695	26.6	0.83	0.65–1.07	52.5	0.88	0.71–1.11
*P*_heterogeneity_				0.0574			0.160
**At least one vision problem diagnosed**							
No	6666	31.3	1.00		55.4	1.00	
Yes	849	29.8	0.94	0.73–1.20	59.0	1.15	0.91–1.44
*P*_heterogeneity_				0.510			0.119
**Recurrence**							
No	6732	31.1	1.00		55.9	1.00	
Yes	919	29.6	0.94	0.76–1.16	54.2	0.96	0.79–1.18
*P*_heterogeneity_				0.449			0.639

a Includes all survivors who are not a student and provided sufficient information on current or most recent employment to enable classification to one of the NSSEC occupational levels.

b For all factors in the above table, with the exception of the treatment factors (surgery, chemotherapy and radiotherapy), the multivariable logistic regression included all factors without surgery, chemotherapy and radiotherapy and the resulting odds ratios and *p* values are from this model. For the treatment factors, the multivariable logistic regression included all factors with the exception of cancer type and the resulting odds ratios and *p* values for the treatment factors are from this model.

c On using the False Discovery Rate method the corrected *p* values indicated that the null hypothesis was not rejected for this particular test of non‐linearity.

#### Non‐manual including managerial/professional and intermediate level occupations

Being classified to a non‐manual occupation was associated with: sex; current age; cancer type; radiotherapy; age at diagnosis and diagnosis with epilepsy (Table 3). For this occupational level, in contrast to when the managerial/professional level was considered separately, females were more likely (OR (99% CI): 1.15 (1.01–1.31)) than males to be classified as non‐manual. Survivors aged under 20 years at questionnaire completion were least likely to be classified as non‐manual, whilst there was little variation after 25 years. All diagnoses, with the exception of leukaemia and Hodgkin's lymphoma, were more likely than CNS neoplasm survivors to be classified to non‐manual. Survivors treated with cranial irradiation were less likely (OR (99% CI): 0.63 (0.52–0.78)) than those who received no radiotherapy to be classified as non‐manual. Survivors diagnosed at 10–14 years were more likely (OR (99% CI): 1.47 (1.06–2.04)) to be classified as non‐manual than those diagnosed below one year. Survivors with epilepsy were less likely (OR (99% CI): 0.70 (0.54–0.89)) to be classified to non‐manual than those without such a diagnosis.

### External analysis

#### Employment status

For survivors overall, there was no evidence of any difference to the general population for being either a student or unemployed (Table 2). However, deficits in comparisons with general population data were observed for all survivors for both working (OR (99% CI): 0.89 (0.81–0.98)) and caring for home/family (0.63 (0.53–0.74)). A considerable excess compared to expected (OR (99% CI): 4.99 (4.06–6.13)) was observed for survivors being unable to work due to illness/disability.

When employment status was considered by diagnosis, with the exception of Hodgkin's lymphoma, all other diagnoses were at increased risk of being unable to work due to illness/disability. The highest odd ratios were observed among CNS neoplasm survivors treated with (OR (99% CI): 15.40 (11.94–19.85)) and without (8.29 (6.08–11.31)) cranial irradiation, but even for non‐Hodgkin's lymphoma survivors who had the lowest increased risk, the OR was still 2.2‐fold (99% CI: 1.34–3.75) compared to the general population. When the deficits for working observed among the whole cohort were broken down by diagnosis, it became evident that the only group with a deficit was CNS neoplasm. The OR was 34% that expected for those treated with cranial irradiation and 64% that expected for those who received no radiotherapy. Cranially irradiated CNS neoplasm survivors were also the only group with an increased likelihood (OR (99% CI): 1.50 (1.04–2.18)) of unemployment compared to the general population. However, on using the FDR correction this association was not statistically significant. Deficits in the proportion caring for home/family were observed for leukaemia (OR (99% CI): 0.60 (0.45–0.82)) and CNS neoplasm survivors (0.45 (0.29–0.71)) treated with radiotherapy and also for Wilms' tumour (0.65 (0.43–0.98)) (although on FDR correction this later association was not statistically significant) and soft tissue sarcoma survivors (0.54 (0.32–0.92)). Although no difference was observed for being a student when survivors were considered overall, differences were observed for specific diagnoses: leukaemia survivors treated with radiotherapy were found to be less likely (OR (99% CI): 0.78 (0.61–0.99)) (although on FDR correction this was not statistically significant) than expected to be a student; and neuroblastoma (1.64 (1.15–2.34)), retinoblastoma (1.50 (1.10–2.05)) and Wilms’ tumour survivors (1.40 (1.07–1.84)) were more likely than expected to be a student. Also survivors of Hodgkin's lymphoma (OR (99% CI): 1.55 (1.18–2.04)), non‐Hodgkin's lymphoma (1.39 (1.04–1.85)) and the ‘other’ neoplasm Group (1.30 (1.04–1.64)) were more likely than the general population to be in employment.

#### Occupational level

Overall survivors were less likely than expected to be classified to a managerial/professional occupation (OR (99% CI): 0.85 (0.77–0.94)) but there was no evidence of a difference between survivors and the general population for non‐manual occupations (Table 4).

**Table 4:** Odds ratios (OR) and 99% confidence intervals (CI) for adult survivors of childhood cancer being classified to a managerial or professional occupational level and also being classified to non‐manual occupational level using the National Statistics Socio‐economic Classification (NSSEC) compared to the general population of Britain, overall and by childhood cancer type, survivors treated with and without radiotherapy are considered separately for CNS neoplasms^a^

	Those classified to the managerial/Professional occupational level^d^			Those classified to the non‐manual occupational level^d^		
Cancer type^a^	OR	(99% CI)	*p* value	OR	(99% CI)	*p* value
All cancer types	0.85	(0.77–0.94)	<0.0005	1.03	(0.93–1.13)	0.471
Leukaemia^c^RT=Yes	0.68	(0.56–0.82)	<0.0005	0.80	(0.67–0.94)	<0.0005
Hodgkin's lymphoma	0.95	(0.76–1.20)	0.589	1.00	(0.80–1.24)	0.956
Non‐Hodgkin's lymphoma	0.94	(0.71–1.23)	0.540	1.18	(0.91–1.54)	0.105
CNS neoplasm^b^ RT=Yes	0.41	(0.32–0.52)	<0.0005	0.57	(0.46–0.70)	<0.0005
RT=No	0.59	(0.45–0.78)	<0.0005	0.89	(0.69–1.13)	0.203
Neuroblastoma	1.08	(0.75–1.56)	0.589	1.24	(0.87–1.77)	0.119
Retinoblastoma	1.19	(0.92–1.53)	0.076	1.55	(1.20–2.01)	<0.0005
Wilms tumour	0.96	(0.77–1.21)	0.682	1.23	(0.98–1.53)	0.017
Bone sarcomas	1.37	(1.01–1.85)	0.008^e^	1.90	(1.37–2.62)	<0.0005
Soft tissue sarcomas	1.14	(0.89–1.46)	0.177	1.20	(0.95–1.53)	0.047
Other neoplasm	1.18	(0.95–1.48)	0.049	1.62	(1.30–2.03)	<0.0005

a Population data from the GHS (2002) was used for the reference group.

b Excludes survivors for whom no record is available regarding radiotherapy.

c There were insufficient leukaemia survivors known to have been unexposed to cranial irradiation for separate meaningful assessment.

d Excludes students and unemployed individuals but includes all other individuals who provided sufficient employment information to classify them according to NSSEC.

e On using the False Discovery Rate method this association was no longer statistically significant.

When considered by diagnosis it became clear that the deficits observed in managerial/professional occupations were limited to cranially irradiated leukaemia survivors (OR (99% CI): 0.68 (0.56–0.82)) and CNS neoplasm survivors treated with (0.41 (0.32–0.52)) or without radiotherapy (0.59 (0.45–0.78)). In fact bone sarcoma survivors were more likely (OR (99% CI): 1.37 (1.01–1.85)) than expected to be in managerial/professional occupations; although on FDR correction this association was not statistically significant. With regard to classification to a non‐manual occupation deficits compared to that expected were observed for both leukaemia (OR (99% CI): 0.80 (0.67–0.94)) and CNS neoplasm survivors treated with cranial irradiation (0.57 (0.46–0.70)). In contrast, for survivors of retinoblastoma (OR (99% CI): 1.55 (1.20–2.01)), bone sarcoma (1.90 (1.37–2.62)) and the ‘other’ neoplasm Group (1.62 (1.30–2.03)) an excess compared to that expected was observed.

## Discussion

One of the most striking findings of this the first, large‐scale and population‐based study to consider employment status in detail among adult survivors of childhood cancer in Britain, relates to the increased likelihood, compared to the general population, of survivors being unable to work due to illness or disability. The odds for survivors overall were five times that expected and when considered by specific cancer, Hodgkin's lymphoma survivors were the only group without an excess, with the odds for CNS neoplasm survivors being 15 times expected for those treated with radiotherapy and 8 times expected for those not receiving radiotherapy.

To our knowledge, only one other population‐based study of 1,716 childhood cancer survivors (born between 1963 and 1976 and aged 26–39 years at time of study) in Sweden has included both a wide spectrum of cancer types and a measure of being unable to work due to illness or disability.^11^ They reported that the proportion receiving economic compensation due to disability among cancer survivors overall was five times that expected from the general population (15% *vs*. 3%); the lowest proportion was observed among lymphoma survivors (4.5%); the highest proportion was observed among CNS neoplasm survivors (28.3%). These findings were generally confirmed by the BCCSS.

In a French multicentre study which included 2,066 childhood cancer survivors (with limited leukaemia survivors), who were treated between 1948 and 2000 and aged between 25 and 64 years at time of study, a significantly higher level of unemployment because of health was reported at 6.5% compared to 4.2% expected from the general population.^5^ Again CNS neoplasm survivors had a significantly higher proportion who were unemployed due to their health (28.1%) compared to that expected (4.3%). However, in the French study, there was no evidence of a difference between the expected and observed proportions for survivors of Hodgkin's lymphoma, bone or soft tissue sarcoma, leukaemia and other diagnoses in relation to unemployment because of health. In the BCCSS survivors of each specific type of childhood cancer were significantly more likely to be unemployed due to illness/disability than expected from the general population, with the exception of Hodgkin's lymphoma. However, the BCCSS is population‐based meaning there is much less potential for confounding influences to bias comparisons between observed and expected.

The Childhood Cancer Survivor Study (CCSS) in North America,^4^ although not population‐based included 6,339 survivors with a variety of diagnoses taken from multiple centres (treated between 1970 and 1986 and aged 25–54 years at study). In a comparison of survivors to their siblings, the CCSS reported a broadly similar excess of survivors being unable to work due to illness/disability (relative risk (95% CI): 6.07 (4.32–8.53)) compared to that seen in the BCCSS general population comparison (OR (99% CI): 4.99 (4.06–6.13)). The likelihood of being unable to work due to illness/disability was significantly increased for each specific cancer type in the CCSS including Hodgkin's lymphoma whereas the BCCSS found no evidence of a difference for Hodgkin's lymphoma.

Several smaller studies of childhood cancer survivors have included varying types of measure for survivors being unable to work due to illness or disability.^12^, ^13^, ^14^, ^15^, ^16^, ^17^ All of these studies found an increased likelihood among survivors when compared to controls for being unable to work due to illness/disability which is consistent with our findings.

The CCSS is the largest study other than the current study to have considered individuals who were unemployed and looking for work as a separate group.^4^ In contrast to the BCCSS which found no difference between survivors and the general population (OR (99% CI): 0.89 (0.72–1.09)), the CCSS found that survivors overall had a higher risk of being unemployed and seeking work (RR (95% CI): 1.90 (1.43–2.54)) and when different cancer types were considered separately the only cancers not at an increased risk compared to siblings were Hodgkin's lymphoma, neuroblastoma and soft tissue sarcoma. Although not as large, the French study,^5^ actually reported a significantly lower proportion of survivors unemployed and seeking work (7.1%) than in the general population (9.5%); and by diagnosis type this significant lower prevalence was also seen for the diagnosis group which included nephroblastoma, neuroblastoma, non‐Hodgkin's lymphoma, retinoblastoma and thyroid tumours (6.6% *vs*. 9.5%). Differences between countries in overall unemployment in childhood cancer survivors have been reported from a meta‐analysis^3^; for U.S. studies unemployment was three times higher (OR (95% CI): 3.24 (2.16–4.86)) than in control groups whereas for the European studies no difference in unemployment was observed (OR (95% CI): 1.00 (0.58–1.70)). Besides different treatment protocols between countries and the subsequent potentially varying risks of late effects for childhood cancer survivors, economic differences in obtaining health care and varying unemployment benefits in different countries could contribute to these differences seen.

When the proportion of BCCSS survivors in employment was compared to the general population of Britain a deficit was observed. However when this was investigated in more detail it was found that the deficit was in fact restricted to only CNS neoplasm survivors. It is reassuring that for all other diagnostic groups either no difference to that expected was observed (cranially irradiated leukaemia survivors; survivors of neuroblastoma, retinoblastoma, Wilms', bone or soft tissue sarcoma) or for three groups (Hodgkin's lymphoma; non‐Hodgkin's lymphoma; the ‘other’ neoplasm group) an increased chance of employment compared to expected was observed. The population‐based study conducted in Sweden^11^ that looked at employment status among adult survivors of childhood cancer by diagnostic group (leukaemia/lymphoma; CNS neoplasm; other cancers) also found that the only difference between survivors and controls was for CNS neoplasm survivors with a deficit of employment compared to controls (85% expected). In the French multicentre study^5^ again a significantly lower proportion of CNS survivors were employed (53.9%) than the general population (79.0%), and no significant difference in employment between observed and expected population rates was seen for survivors of Hodgkin's lymphoma, bone or soft tissue sarcomas, leukaemia and the ‘other’ diagnosis group.

When occupational level was considered among BCCSS survivors overall, although survivors were less likely than expected (OR (99% CI): 0.85 (0.77–0.94)) to have a managerial/professional occupation, when individuals classified to intermediate level occupations were also included and compared to the group of routine/manual occupations there was no evidence of a difference between survivors and the general population of Britain (1.03 (0.93–1.13)). The investigations by diagnostic group revealed that the deficit observed for survivors being classified to managerial/professional occupations was restricted to leukaemia survivors treated with cranial irradiation and survivors of CNS neoplasm treated either with or without radiotherapy. It is encouraging that for the other diagnostic groups there was no evidence of a difference from that expected in terms of managerial/professional occupation classification with the exception of bone sarcoma survivors who were in fact found to have an increased likelihood over that expected (OR (99% CI): 1.37 (1.01–1.85)). However, caution should be exercised in interpreting this last statement in relation to bone sarcoma survivors since on FDR correction this association was no longer statistically significant. With regard to the proportion in non‐manual occupations deficits were again observed for cranially irradiated leukaemia and CNS neoplasm survivors. However, reassuringly, for other diagnostic groups there was either no evidence of a difference or for some groups (retinoblastoma, bone sarcoma, other neoplasm) an excess was observed for survivors compared to the general population.

In the BCCSS compared to the general population, survivors overall were less likely to be in the highest occupational level (managerial/professional). However, in the French study^5^ they reported that overall significantly more survivors (23.1%) than expected (15.4%) were in professional and managerial occupations; and this excess was also seen in the bone and soft tissue sarcoma survivor Group (27.7% *vs*. 15.9%) and for the ‘other’ diagnosis group which included retinoblastoma (24.7% *vs*. 15.3%). Although the French study did report a significant deficit in professional occupations in CNS neoplasm survivors (6.2% *vs*. 15.6%) as was seen in the BCCSS. In the CCSS,^6^ all survivors were less likely to be in professional occupations than siblings to a similar degree (RR (95% CI): 0.93 (0.89–0.98)) as that reported in the BCCSS general population comparison. By cancer type, the CCSS found that leukaemia, non‐Hodgkin's lymphoma as well as CNS neoplasm survivors reported significantly fewer professional occupations than siblings. Similar to the French study and the BCCSS, bone cancer survivors from the CCSS were more likely to be in a professional occupation than siblings (RR (95% CI): 1.26 (1.03–1.54)).

The BCCSS considered factors related to both the various categories of employment status and occupational level. Similar to the BCCSS, in the French study,^5^ those: treated with cranial irradiation, with a CNS neoplasm, younger at study or who were female were less likely to be managers than the corresponding complementary groups. Also in the CCSS,^6^ survivors: diagnosed at a younger age, with a CNS neoplasm or treated with cranial irradiated were less likely to hold managerial occupations. However in the CCSS, female survivors were in fact more likely to have a managerial occupation than males.

In terms of employment status, we found that female survivors were more likely than male survivors to be caring for home or family and unable to work due to illness or disability but less likely to be in employment and less likely to be unemployed and looking for work. Numerous other studies found that, as in the BCCSS, males were more likely than females to be in employment and less likely to be unemployed due to health.^4^, ^5^, ^18^, ^19^, ^20^, ^21^ In the BCCSS, CNS neoplasm survivors were more likely than the majority of other types of childhood cancer to be unable to work due to illness or disability and they were less likely than survivors of leukaemia, Hodgkin's lymphoma, retinoblastoma or the ‘other’ neoplasm group to be in current employment. From a meta‐analysis on employment in childhood cancer survivors, it was reported that for CNS neoplasm survivors they were five times more likely to be unemployed as adults than controls (OR: 4.7 95 CI: 1.2–18.7).^3^ CNS neoplasm survivors are at a particular increased risk for late effects such as recurrence, progression of their first neoplasm, endocrine, neurological and sensory complications which could all influence employment status; these risks do not abate with period since diagnosis.^22^ In our study treatment with cranial radiotherapy was associated with a decreased likelihood of being in employment and an increased likelihood of being unable to work due to illness or disability. Also younger age at diagnosis decreased the likelihood of being in current employment and increased the likelihood of being a student or being unable to work due to illness or disability. Both cranial irradiation and a young age at treatment are associated with neurocognitive deficits,^23^ which could reduce employment opportunities for such survivors. Previously from the BCCSS, both cranial irradiation and young age at diagnosis were shown to be associated with reduced educational attainment in the survivors.^24^ In addition, health problems that these survivors are known to experience at an increasing level as the survivors age,^25^ could be impeding their employment opportunities. Previous studies that considered diagnosis with specified medical conditions and employment found an association with an increased likelihood of unemployment for: epilepsy/motor impairment^3^; hearing loss, blindness, heart or lung disease, stroke and depression^26^; one or more chronic medical condition^19^; recurrence and a SPT.^4^ In the BCCSS, survivors diagnosed with a SPT, epilepsy, a hearing problem, a visual problem or a recurrence were all less likely than those without such a diagnosis to be in current employment. Survivors diagnosed with a SPT, epilepsy, a hearing/visual problem or a recurrence were more likely than those without such a diagnosis to be unable to work due to illness or disability. In relation to age at questionnaire completion the likelihood of being unable to work due to illness or disability increased with age and the likelihood of employment also increased with age but only until 40 years. The former could be a consequence of the accelerated late effects seen with aging in the survivors.^25^

Current follow‐up guidelines for childhood cancer survivors in Great Britain suggest that survivors diagnosed with cancer and in particular those who received cranial irradiation, were treated at a young age and/or had a CNS neoplasm should have regular cognitive assessment and educational support should be offered as well as support for obtaining employment.^27^, ^28^ Guidelines in the United States recommend yearly psychosocial assessment with particular emphasis on following educational and vocational progress for all types of cancer diagnoses, although the highest risk is noted in CNS neoplasm survivors and those who had CNS directed treatment.^29^ This study has provided evidence of factors associated with employment and occupational level such as epilepsy, hearing or vision problems which could be targeted in interventions and in follow‐up clinics to help survivors achieve their full potential in gaining suitable employment. As suggested by others interventions for childhood cancer survivors should target physical health barriers to employment, as well as screening for mental health and neurocognitive problems.^30^ The Department of Health 2011 Strategy for Cancer^31^ highlights deficiencies in current cancer follow‐up which is failing to meet the psychosocial needs of patients following treatment. As a result, a holistic needs assessment tool has been developed which can be used in the clinical setting, to identify psychological, social, spiritual, financial, employment and educational needs which can then be addressed through support and signposting to appropriate services.^32^

The key strengths of this study were that employment status was available on over 10,000 survivors who had been followed up for a considerable period of time from diagnosis; over a quarter of them were aged 35 years or older at questionnaire completion. The BCCSS is also population‐based and includes most adult survivors of childhood cancer diagnosed in Britain between 1940 and 1991. Other studies have used cohorts of adult survivors of childhood cancer from selected cancer centres which could affect the generalisability of the results to all childhood cancer survivors in the population.

## Conclusion

Obtaining employment and professional occupations is a problem for some groups of childhood cancer survivors, in‐particular for cranially irradiated CNS neoplasm and leukaemia survivors. Several factors were associated with employment status and occupational level such as childhood cancer type, radiotherapy and medical conditions, for example, epilepsy, which could indicate where intervention might be best directed to support survivors in maximising their chance of attaining employment and professional occupations.

## Supporting information

Supporting Information
