# Supplementary material for: Employment status and occupational level of adult survivors of childhood cancer in Great Britain: The British childhood cancer survivor study
Source: Int J Cancer. 2017 Apr 7;140(12):2678–92. doi: 10.1002/ijc.30696 (PMC5434894; doi:10.1002/ijc.30696)
Supplement: Supplementary file 1 — Supporting Information [file IJC-140-2678-s001.doc]

**Supplementary eT**able 1: Demographic, cancer, treatment and medical condition characteristics of the BCCSS population in relation to employment status and occupational level.

|  | **EMPLOYMENT STATUS** | | | | | | | **OCCUPATIONAL LEVEL**  **(National Statistic Socio-economic Group)*** | | |
| --- | --- | --- | --- | --- | --- | --- | --- | --- | --- | --- |
|  | **Employed** | **Unemployed and looking for work** | **Unable to work due to illness or disability** | **Student** | **Caring for home or family** | **Retired** | **TOTAL** | **Managerial / professional** | **Non-manual** | **TOTAL** |
|  | **N (%)** | **N (%)** | **N (%)** | **N (%)** | **N (%)** | **N (%)** | **N** | **N (%)** | **N (%)** | **N** |
| **Total** | 6415 (62.5) | 446 (4.3) | 1094 (10.7) | 1752 (17.1) | 482 (4.7) | 68 (0.7) | 10257 | 2373 (30.8) | 4277 (55.6) | 7699 |
|  |  |  |  |  |  |  |  |  |  |  |
| **Gender** |  |  |  |  |  |  |  |  |  |  |
| Male | 3531 (67.2) | 275 (5.2) | 496 (9.4) | 893 (17.0) | 30 (0.6) | 31 (0.6) | 5256 | 1304 (32.5) | 2137 (53.2) | 4014 |
| Female | 2884 (57.7) | 171 (3.4) | 598 (12.0) | 859 (17.2) | 452 (9.0) | 37 (0.7) | 5001 | 1069 (29.0) | 2140 (58.1) | 3685 |
|  |  |  |  |  |  |  |  |  |  |  |
| **Current age (at questionnaire completion in years)** |  |  |  |  |  |  |  |  |  |  |
| 16 to 19 | 496 (24.9) | 104 (5.2) | 53 (2.7) | 1331 (66.9) | 7 (0.4) | 0 (0.0) | 1991 | 21 (3.8) | 125 (22.7) | 550 |
| 20 to 24 | 1086 (63.4) | 114 (6.7) | 143 (8.4) | 312 (18.2) | 57 (3.3) | 0 (0.0) | 1712 | 288 (23.3) | 605 (49.0) | 1234 |
| 25 to 29 | 1439 (76.7) | 76 (4.1) | 204 (10.9) | 65 (3.5) | 91 (4.9) | 2 (0.1) | 1877 | 598 (36.5) | 987 (60.2) | 1640 |
| 30 to 34 | 1248 (74.8) | 67 (4.0) | 205 (12.3) | 24 (1.4) | 122 (7.3) | 2 (0.1) | 1668 | 511 (34.2) | 893 (59.8) | 1493 |
| 35 to 39 | 946 (75.4) | 38 (3.0) | 165 (13.2) | 11 (0.9) | 94 (7.5) | 1 (0.1) | 1255 | 403 (34.9) | 690 (59.7) | 1156 |
| 40 to 44 | 528 (71.0) | 22 (3.0) | 135 (18.2) | 7 (0.9) | 47 (6.3) | 5 (0.7) | 744 | 254 (37.4) | 420 (61.8) | 680 |
| 45 to 49 | 354 (73.0) | 14 (2.9) | 90 (18.6) | 2 (0.4) | 22 (4.5) | 3 (0.6) | 485 | 150 (33.5) | 274 (61.2) | 448 |
| 50 to 54 | 226 (67.9) | 8 (2.4) | 63 (18.9) | 0 (0.0) | 30 (9.0) | 6 (1.8) | 333 | 90 (28.6) | 179 (56.8) | 315 |
| >= 55 | 92 (47.9) | 3 (1.6) | 36 (18.8) | 0 (0.0) | 12 (6.3) | 49 (25.5) | 192 | 58 (31.7) | 104 (56.8) | 183 |
|  |  |  |  |  |  |  |  |  |  |  |
| **Cancer type** |  |  |  |  |  |  |  |  |  |  |
| CNS neoplasm | 1109 (51.5) | 111 (5.2) | 562 (26.1) | 246 (11.4) | 97 (4.5) | 28 (1.3) | 2153 | 335 (21.9) | 739 (48.4) | 1528 |
| Leukaemia | 1692 (60.0) | 140 (5.0) | 181 (6.4) | 681 (24.2) | 124 (4.4) | 1 (0.04) | 2819 | 485 (25.0) | 923 (47.5) | 1943 |
| Hodgkin's lymphoma | 587 (81.1) | 27 (3.7) | 35 (4.8) | 41 (5.7) | 28 (3.9) | 6 (0.8) | 724 | 247 (37.4) | 382 (57.9) | 660 |
| Non-Hodgkin's lymphoma | 395 (74.5) | 18 (3.4) | 32 (6.0) | 58 (10.9) | 22 (4.2) | 5 (0.9) | 530 | 157 (34.9) | 266 (59.1) | 450 |
| Neuroblastoma | 227 (54.0) | 19 (4.5) | 24 (5.7) | 130 (31.0) | 19 (4.5) | 1 (0.2) | 420 | 91 (33.7) | 156 (57.8) | 270 |
| Retinoblastoma | 434 (62.7) | 40 (5.8) | 49 (7.1) | 130 (18.8) | 34 (4.9) | 5 (0.7) | 692 | 201 (38.1) | 344 (65.2) | 528 |
| Wilms' tumour | 601 (63.0) | 33 (3.5) | 51 (5.4) | 217 (22.8) | 48 (5.0) | 4 (0.4) | 954 | 232 (32.9) | 415 (58.8) | 706 |
| Bone sarcoma | 283 (72.8) | 7 (1.8) | 42 (10.8) | 20 (5.1) | 30 (7.7) | 7 (1.8) | 389 | 148 (42.7) | 249 (71.8) | 347 |
| Soft tissue sarcoma | 474 (67.1) | 25 (3.5) | 61 (8.6) | 113 (16.0) | 27 (3.8) | 6 (0.8) | 706 | 211 (37.9) | 332 (59.6) | 557 |
| Other neoplasm | 613 (70.5) | 26 (3.0) | 57 (6.6) | 116 (13.3) | 53 (6.1) | 5 (0.6) | 870 | 266 (37.5) | 471 (66.3) | 710 |
|  |  |  |  |  |  |  |  |  |  |  |
| **Surgery** |  |  |  |  |  |  |  |  |  |  |
| No | 2366 (70.5) | 152 (4.5) | 309 (9.2) | 342 (10.2) | 175 (5.2) | 11 (0.3) | 3355 | 819 (29.7) | 1477 (53.7) | 2753 |
| Yes | 2850 (68.1) | 153 (3.7) | 629 (15.0) | 251 (6.0) | 246 (5.9) | 56 (1.3) | 4185 | 1216 (34.1) | 2137 (59.9) | 3567 |
| Not known | 1199 (44.1) | 141 (5.2) | 156 (5.7) | 1159 (42.7) | 61 (2.2) | 1 (0.04) | 2717 | 338 (24.5) | 663 (48.1) | 1379 |
|  |  |  |  |  |  |  |  |  |  |  |
| **Radiotherapy** (RT) |  |  |  |  |  |  |  |  |  |  |
| No RT | 1518 (69.8) | 71 (3.3) | 227 (10.4) | 191 (8.8) | 152 (7.0) | 17 (0.8) | 2176 | 627 (34.2) | 1113 (60.6) | 1836 |
| Non-cranial RT | 1710 (76.6) | 68 (3.0) | 213 (9.6) | 98 (4.4) | 113 (5.1) | 29 (1.3) | 2231 | 808 (39.7) | 1321 (64.9) | 2037 |
| Cranial RT | 1820 (62.6) | 159 (5.5) | 480 (16.5) | 290 (10.0) | 143 (4.9) | 17 (0.6) | 2909 | 532 (23.6) | 1069 (47.5) | 2250 |
| Not known | 1367 (46.5) | 148 (5.0) | 174 (5.9) | 1173 (39.9) | 74 (2.5) | 5 (0.2) | 2941 | 406 (25.8) | 774 (49.1) | 1576 |
|  |  |  |  |  |  |  |  |  |  |  |
| **Chemotherapy** |  |  |  |  |  |  |  |  |  |  |
| No | 2158 (66.0) | 119 (3.6) | 560 (17.1) | 168 (5.1) | 200 (6.1) | 63 (1.9) | 3268 | 877 (31.7) | 1625 (58.8) | 2765 |
| Yes | 2738 (71.4) | 167 (4.4) | 308 (8.0) | 417 (10.9) | 201 (5.2) | 3 (0.1) | 3834 | 1021 (32.3) | 1765 (55.8) | 3164 |
| Not known | 1519 (48.1) | 160 (5.1) | 226 (7.2) | 1167 (37.0) | 81 (2.6) | 2 (0.1) | 3155 | 475 (26.8) | 887 (50.1) | 1770 |
|  |  |  |  |  |  |  |  |  |  |  |
| **Age at cancer diagnosis (years)** |  |  |  |  |  |  |  |  |  |  |
| 0 | 439 (52.6) | 34 (4.1) | 63 (7.6) | 261 (31.3) | 35 (4.2) | 2 (0.2) | 834 | 190 (36.3) | 315 (60.1) | 524 |
| 1 to 4 | 2103 (53.9) | 195 (5.0) | 353 (9.1) | 1101 (28.2) | 139 (3.6) | 9 (0.2) | 3900 | 621 (25.0) | 1222 (49.2) | 2484 |
| 5 to 9 | 1815 (66.8) | 130 (4.8) | 315 (11.6) | 317 (11.7) | 129 (4.7) | 13 (0.5) | 2719 | 609 (28.0) | 1143 (52.6) | 2174 |
| 10 to 14 | 2058 (73.4) | 87 (3.1) | 363 (13.0) | 73 (2.6) | 179 (6.4) | 44 (1.6) | 2804 | 953 (37.9) | 1597 (63.4) | 2517 |
|  |  |  |  |  |  |  |  |  |  |  |
| **Second primary tumour** |  |  |  |  |  |  |  |  |  |  |
| No | 6064 (62.9) | 422 (4.4) | 962 (10.0) | 1700 (17.6) | 446 (4.6) | 54 (0.6) | 9648 | 2215 (30.7) | 3987 (55.3) | 7209 |
| Yes | 351 (57.6) | 24 (3.9) | 132 (21.7) | 52 (8.5) | 36 (5.9) | 14 (2.3) | 609 | 158 (32.2) | 290 (59.2) | 490 |
|  |  |  |  |  |  |  |  |  |  |  |
| **Epilepsy or repeated seizures/fits** |  |  |  |  |  |  |  |  |  |  |
| No | 5944 (65.8) | 379 (4.2) | 660 (7.3) | 1559 (17.3) | 436 (4.8) | 52 (0.6) | 9030 | 2244 (32.1) | 3950 (56.6) | 6982 |
| Yes | 374 (38.2) | 53 (5.4) | 373 (38.1) | 132 (13.5) | 35 (3.6) | 13 (1.3) | 980 | 109 (18.6) | 266 (45.4) | 586 |
| Not known | 97 (39.3) | 14 (5.7) | 61 (24.7) | 61 (24.7) | 11 (4.5) | 3 (1.2) | 247 | 20 (15.3) | 61 (46.6) | 131 |
|  |  |  |  |  |  |  |  |  |  |  |
| **At least one hearing problem** |  |  |  |  |  |  |  |  |  |  |
| No | 5756 (63.9) | 385 (4.3) | 816 (9.1) | 1570 (17.4) | 432 (4.8) | 43 (0.5) | 9002 | 2153 (31.6) | 3829 (56.3) | 6805 |
| Yes | 512 (53.1) | 42 (4.4) | 221 (22.9) | 136 (14.1) | 34 (3.5) | 20 (2.1) | 965 | 185 (26.6) | 365 (52.5) | 695 |
| Not known | 147 (50.7) | 19 (6.6) | 57 (19.7) | 46 (15.9) | 16 (5.5) | 5 (1.7) | 290 | 35 (17.6) | 83 (41.7) | 199 |
|  |  |  |  |  |  |  |  |  |  |  |
| **At least one vision problem by age at diagnosis group** |  |  |  |  |  |  |  |  |  |  |
| No | 5690 (65.5) | 353 (4.1) | 693 (8.0) | 1483 (17.1) | 413 (4.8) | 52 (0.6) | 8684 | 2084 (31.3) | 3693 (55.4) | 6666 |
| Yes | 592 (45.6) | 76 (5.9) | 342 (26.4) | 218 (16.8) | 54 (4.2) | 15 (1.2) | 1297 | 253 (29.8) | 501 (59.0) | 849 |
| Not known | 133 (48.2) | 17 (6.2) | 59 (21.4) | 51 (18.5) | 15 (5.4) | 1 (0.4) | 276 | 36 (19.6) | 83 (45.1) | 184 |
|  |  |  |  |  |  |  |  |  |  |  |
| **Recurrence** |  |  |  |  |  |  |  |  |  |  |
| No | 5669 (64.1) | 373 (4.2) | 842 (9.5) | 1481 (16.7) | 422 (4.8) | 61 (0.7) | 8848 | 2095 (31.1) | 3765 (55.9) | 6732 |
| Yes | 711 (53.3) | 67 (5.0) | 236 (17.7) | 260 (19.5) | 54 (4.0) | 6 (0.4) | 1334 | 272 (29.6) | 498 (54.2) | 919 |
| Not known | 35 (46.7) | 6 (8.0) | 16 (21.3) | 11 (14.7) | 6 (8.0) | 1 (1.3) | 75 | 6 (12.5) | 14 (29.2) | 48 |

* including all who are not a student and who could be allocated to an occupational level code by current or most recent employment information

**Supplementary eTable 2: Frequency of survivors for the outcomes being: a student;** or caring for home or family, and the corresponding ORs (99% CIs) from multivariable logistic regression for these outcomes with selected demographic, cancer and health related factors in the childhood cancer survivors.

| **Factor** | **Total number**  **of survivors** | **Survivors who were a student** | | | **Survivors who were caring for home or family** | | |
| --- | --- | --- | --- | --- | --- | --- | --- |
|  |  | **% of survivors who were a student** | **Adjusted odds**  **ratio for being a student vs not*** | **99% CI** | **% of survivors who were caring for the home or family** | **Adjusted odds**  **ratio for caring for home or family vs not*** | **99% CI** |
| **Gender** |  |  |  |  |  |  |  |
| Male | 5256 | 17.0 | 1.00 |  | 0.6 | 1.00 |  |
| Female | 5001 | 17.2 | 1.16 | 0.96 - 1.41 | 9.0 | 18.25 | 10.78 – 30.92 |
| P_heterogeneity_ |  |  |  | 0.043 |  |  | <0.001 |
|  |  |  |  |  |  |  |  |
| **Current age (at questionnaire completion in years)** |  |  |  |  |  |  |  |
| 16 – 19 | 1991 | 66.9 | 1.00 |  | 0.4 | 1.00 |  |
| 20 – 24 | 1712 | 18.2 | 0.12 | 0.10 - 0.15 | 3.3 | 9.56 | 3.11 – 29.36 |
| 25 – 29 | 1877 | 3.5 | 0.02 | 0.01 - 0.03 | 4.9 | 15.63 | 5.19 – 47.11 |
| 30 – 34 | 1668 | 1.4 | 0.01 | 0.004 - 0.01 | 7.3 | 24.30 | 8.11 – 72.81 |
| 35 – 39 | 1255 | 0.9 | 0.01 | 0.002 - 0.01 | 7.5 | 22.27 | 7.33 – 67.73 |
| 40 – 44 | 744 | 0.9 | 0.005 | 0.002 - 0.01 | 6.3 | 22.63 | 7.17 – 71.49 |
| 45 – 49 | 485 | 0.4 | 0.001 | 0.000 – 0.02 | 4.5 | 15.30 | 4.45 – 52.65 |
| 50 – 54 | 333 | 0.0 | N/A | N/A | 9.0 | 30.44 | 9.06 – 102.23 |
| ≥ 55 | 192 | 0.0 | N/A | N/A | 6.3 | 21.01 | 5.20 – 84.90 |
| P_heterogeneity_ |  |  |  | <0.001 |  |  | <0.001 |
| P _linear trend_ (P _non-linearity_) |  |  |  | <0.001 (<0.001) |  |  | <0.001 (<0.001) |
|  |  |  |  |  |  |  |  |
| **Cancer type** |  |  |  |  |  |  |  |
| CNS neoplasm | 2153 | 11.4 | 1.00 |  | 4.5 | 1.00 |  |
| Leukaemia | 2819 | 24.2 | 0.89 | 0.65 – 1.23 | 4.4 | 1.18 | 0.77 – 1.82 |
| Hodgkin's lymphoma | 724 | 5.7 | 1.10 | 0.61 - 2.00 | 3.9 | 0.86 | 0.45 – 1.63 |
| Non-hodgkin's lymphoma | 530 | 10.9 | 0.95 | 0.54 – 1.65 | 4.2 | 1.01 | 0.50 – 2.06 |
| Neuroblastoma | 420 | 31.0 | 1.12 | 0.67 - 1.88 | 4.5 | 1.12 | 0.50 – 2.49 |
| Retinoblastoma | 692 | 18.8 | 0.96 | 0.58 – 1.61 | 4.9 | 1.01 | 0.50 - 2.03 |
| Wilms' tumour | 954 | 22.8 | 1.20 | 0.80 – 1.80 | 5.0 | 1.11 | 0.63 – 1.97 |
| Bone sarcoma | 389 | 5.1 | 0.78 | 0.36 – 1.67 | 7.7 | 1.27 | 0.68 – 2.38 |
| Soft tissue sarcomas | 706 | 16.0 | 1.00 | 0.62 – 1.60 | 3.8 | 0.80 | 0.42 – 1.52 |
| Other neoplasm | 870 | 13.3 | 0.90 | 0.57 - 1.42 | 6.1 | 0.97 | 0.58 - 1.64 |
| P_heterogeneity_ |  |  |  | 0.546 |  |  | 0.810 |
|  |  |  |  |  |  |  |  |
| **Treatment** |  |  |  |  |  |  |  |
| Surgery No | 3355 | 10.2 | 1.00 |  | 5.2 | 1.00 |  |
| Yes | 4185 | 6.0 | 1.13 | 0.74 - 1.73 | 5.9 | 0.87 | 0.59 - 1.28 |
| P_heterogeneity_ |  |  |  | 0.444 |  |  | 0.358 |
|  |  |  |  |  |  |  |  |
| Radiotherapy (RT) No RT | 2176 | 8.8 | 1.00 |  | 7.0 | 1.00 |  |
| Non-cranial RT | 2231 | 4.4 | 0.96 | 0.61 - 1.52 | 5.1 | 0.64 | 0.44 - 0.94 |
| Cranial RT | 2909 | 10.0 | 0.94 | 0.61 - 1.46 | 4.9 | 0.80 | 0.53 - 1.21 |
| P_heterogeneity_ |  |  |  | 0.939 |  |  | 0.0110 |
|  |  |  |  |  |  |  |  |
| Chemotherapy No | 3268 | 5.1 | 1.00 |  | 6.1 | 1.00 |  |
| Yes | 3834 | 10.9 | 1.08 | 0.72 - 1.63 | 5.2 | 0.96 | 0.65 - 1.42 |
| P_heterogeneity_ |  |  |  | 0.619 |  |  | 0.774 |
|  |  |  |  |  |  |  |  |
| **Age at cancer diagnosis (years)** |  |  |  |  |  |  |  |
| 0 | 834 | 31.3 | 1.00 |  | 4.2 | 1.00 |  |
| 1 – 4 | 3900 | 28.2 | 0.81 | 0.57 – 1.14 | 3.6 | 0.79 | 0.44 - 1.43 |
| 5 – 9 | 2719 | 11.7 | 0.54 | 0.36 - 0.80 | 4.7 | 0.93 | 0.48 – 1.78 |
| 10 – 14 | 2804 | 2.6 | 0.57 | 0.34 - 0.95 | 6.4 | 0.97 | 0.50 – 1.88 |
| P_heterogeneity_ |  |  |  | <0.001 |  |  | 0.487 |
| P_linear trend_ (P_non-linearity_) |  |  |  | <0.001 (0.110) |  |  | 0.427 (0.405) |
|  |  |  |  |  |  |  |  |
| **Second primary tumour diagnosed** |  |  |  |  |  |  |  |
| No | 9648 | 17.6 | 1.00 |  | 4.6 | 1.00 |  |
| Yes | 609 | 8.5 | 1.59 | 0.92 – 2.74 | 5.9 | 0.87 | 0.52 - 1.46 |
| P_heterogeneity_ |  |  |  | 0.034 |  |  | 0.491 |
|  |  |  |  |  |  |  |  |
| **Epilepsy or repeated seizures/fits diagnosed** |  |  |  |  |  |  |  |
| No | 9030 | 17.3 | 1.00 |  | 4.8 | 1.00 |  |
| Yes | 980 | 13.5 | 0.85 | 0.59 - 1.24 | 3.6 | 0.69 | 0.41 - 1.17 |
| P_heterogeneity_ |  |  |  | 0.272 |  |  | 0.061 |
|  |  |  |  |  |  |  |  |
| **At least one hearing problem diagnosed** |  |  |  |  |  |  |  |
| No | 9002 | 17.4 | 1.00 |  | 4.8 | 1.00 |  |
| Yes | 965 | 14.1 | 0.93 | 0.65 – 1.35 | 3.5 | 0.81 | 0.49 - 1.35 |
| P_heterogeneity_ |  |  |  | 0.628 |  |  | 0.275 |
|  |  |  |  |  |  |  |  |
| **At least one vision problem diagnosed** |  |  |  |  |  |  |  |
| No | 8684 | 17.1 | 1.00 |  | 4.8 | 1.00 |  |
| Yes | 1297 | 16.8 | 1.26 | 0.89 – 1.79 | 4.2 | 0.82 | 0.51 - 1.33 |
| P_heterogeneity_ |  |  |  | 0.086 |  |  | 0.293 |
|  |  |  |  |  |  |  |  |
| **Recurrence** |  |  |  |  |  |  |  |
| No | 8848 | 16.7 | 1.00 |  | 4.8 | 1.00 |  |
| Yes | 1334 | 19.5 | 1.27 | 0.96 - 1.68 | 4.0 | 0.94 | 0.62 - 1.43 |
| P_heterogeneity_ |  |  |  | 0.029 |  |  | 0.707 |

*- For all factors in the above table, with the exception of the treatment factors (surgery, chemotherapy and radiotherapy), the multivariable logistic regression included all factors without surgery, chemotherapy and radiotherapy, and the resulting odds ratios and p values are from this model. For the treatment factors, the multivariable logistic regression included all factors with the exception of cancer type and the resulting odds ratios and p values for the treatment factors are from this model.
